# Effective production of human growth factors in *Escherichia coli* by fusing with small protein 6HFh8

**DOI:** 10.1186/s12934-020-01502-1

**Published:** 2021-01-07

**Authors:** Young Su Kim, Hye-Jeong Lee, Man-ho Han, Nam-kyung Yoon, Yeu-chun Kim, Jungoh Ahn

**Affiliations:** 1 Department of Chemical and Biomolecular Engineering, KAIST, Daejeon, 34141 Republic of Korea; 2 Biotechnology Process Engineering Center, KRIBB, Cheongju, 28116 Republic of Korea; 3 Department of Bioprocess Engineering, KRIBB School of Biotechnology, Korea University of Science and Technology (UST), 217 Gajeong-ro, Yuseong-gu, Daejeon, 34113 Republic of Korea

**Keywords:** Human derived growth factor, aFGF, VEGF, *Escherichia coli*, Fh8 from *Fasciola hepatica*

## Abstract

**Background:**

Growth factors (GFs) are signaling proteins that affect cellular processes such as growth, proliferation, and differentiation. GFs are used as cosmeceuticals, exerting anti-wrinkle, anti-aging, and whitening effects, and also as pharmaceuticals to treat wounds, growth failure, and oral mucositis. However, in mammalian and bacterial cells, low productivity and expression in inclusion bodies, respectively, of GFs does not satisfy the consumer demand. Here, we aimed to develop a bacterial expression system that produces high yields of soluble GFs that can be purified in their native forms.

**Results:**

We present Fh8, an 8-kDa peptide from *Fasciola hepatica* with an N-terminal hexa-histidine (6HFh8), as a fusion partner for enhanced human GF production in recombinant *Escherichia coli*. The fusion partner harboring a tobacco etch virus (TEV) protease cleavage site was fused to the N-terminus of 10 human GFs: acidic and basic fibroblast growth factors (aFGF and bFGF, respectively), epidermal growth factor (EGF), human growth hormone (hGH), insulin-like growth factor 1 (IGF-1), vascular endothelial growth factor 165 (VEGF165), keratinocyte growth factor 1 (KGF-1), placental growth factor (PGF), stem cell factor (SCF), and tissue inhibitor of metalloproteinase 1 (TIMP-1). The fusion proteins were expressed in *E. coli* under the control of T7 promoter at three temperatures (25 °C, 30 °C, and 37 °C). All individual fusion proteins, except for SCF and TIMP-1, were successfully overexpressed in cytoplasmic soluble form at more than one temperature. Further, the original aFGF, IGF-1, EGF, and VEGF165 proteins were cleaved from the fusion partner by TEV protease. Five-liter fed-batch fermentation approaches for the 6HFh8-aFGF (lacking disulfide bonds) and 6HFh8-VEGF165 (a cysteine-rich protein) were devised to obtain the target protein at concentrations of 9.7 g/l and 3.4 g/l, respectively. The two GFs were successfully highly purified (> 99% purity). Furthermore, they exerted similar cell proliferative effects as those of their commercial equivalents.

**Conclusions:**

We demonstrated that 6HFh8-GF fusion proteins could be overexpressed on a g/l scale in the cytoplasm of *E. coli*, with the GFs subsequently highly purified and maintaining their biological activity. Hence, the small protein 6HFh8 can be used for efficient mass-production of various GFs.

## Background

Growth factors (GFs) are signaling proteins that positively regulate various cellular processes by binding appropriate receptors on the cell surface. The signaling pathways induced by GFs regulate cellular responses, such as growth, differentiation, proliferation, maintenance, inflammation, and angiogenesis [1, 2]. Traditionally, epidermal GF (EGF), vascular endothelial GF (VEGF), keratinocyte GF (KGF), insulin-like GF (IGF), and fibroblast GF (FGF) family members are used for wound healing, curing and targeting diseases, and stimulating hair growth [3–10]. Currently, because their roles in cellular regulation in vivo include anti-wrinkle, anti-aging, anti-hair loss, and tissue repair effects, GFs are also used as cosmetic additives [11–14]. Owing to the increase in the demand for GFs, various expression systems, including *Escherichia coli* [1, 15–19], *Bacillus subtilis* [20], mammalian cell [21], baculovirus [22], silkworm [23, 24], and plant [11, 12] systems, are used for cost-effective and efficient production of recombinant GFs. Among them, the *E. coli* system is frequently used for the production of recombinant proteins because of its facile genetic modification, rapid protein expression, and high growth rate. However, there are several limitations to this system, such as protein expression in inclusion bodies, incorrect folding, and inactive protein production caused by mispairing of disulfide bonds [25]. These limitations lead to a low production yield [26, 27].

To overcome the mispairing of disulfide bonds, *E. coli* host strains have been genetically engineered to control the cellular redox environment [26–28]. As another strategy, solubility enhancers, such as maltose-binding protein (MBP) [1, 17], oleosin [12], low-molecular-weight protamine [14], HaloTag [18], glutathione *S*-transferase [1, 17], protein disulfide isomerase (PDI) [1, 17], ELK16 [29], and thioredoxin [1, 17, 30], are fused to the target protein, including GFs. As the solubility of the tag affects the solubility of the passenger protein, choosing an appropriate solubility tag is critical [31]. Nguyen et al. [1, 17] reported that seven protein tags, namely, thioredoxin, glutathione *S*-transferase, N-utilization substance protein A, the b’a’domain of PDI (PDIb’a’), PDI, 6×His, and MBP, were tested, and some or all of the protein tags enhance the solubility of the GFs in *E. coli*. The fusion with MBP showed the best solubility, but the production yield was low (2.9%). In general, if the fusion partner is larger than the target protein, it solubilizes the fusion protein by overriding the target protein solubility. In that case, removal of the fusion partner may negatively influence the solubility and stability of the target protein. However, the use of a small fusion partner allows a reliable assessment of the target protein behavior [32], improving the prediction of protein yield after tag removal. Therefore, when using a fusion partner with a size that is similar to or smaller than that of the GFs, it is necessary to test how they are expressed in the *E. coli* system.

Fh8 protein, an antigen secreted by *Fasciola hepatica*, has been used to diagnose fascioliasis [33, 34]. Costa et al. [32, 35] demonstrated that Fh8, an 8-kDa protein, and Fh8 with an N-terminal hexa-histidine (6HFh8) could be used as a fusion partner that increases the solubility of various target proteins to a greater extent than other protein tags. Further, we showed that using 6HFh8 as a fusion partner facilitates the solubilization of N-terminal pro-brain natriuretic peptide, which increases approximately 97.5-fold in the final product yield [38].

**Fig. 1:**
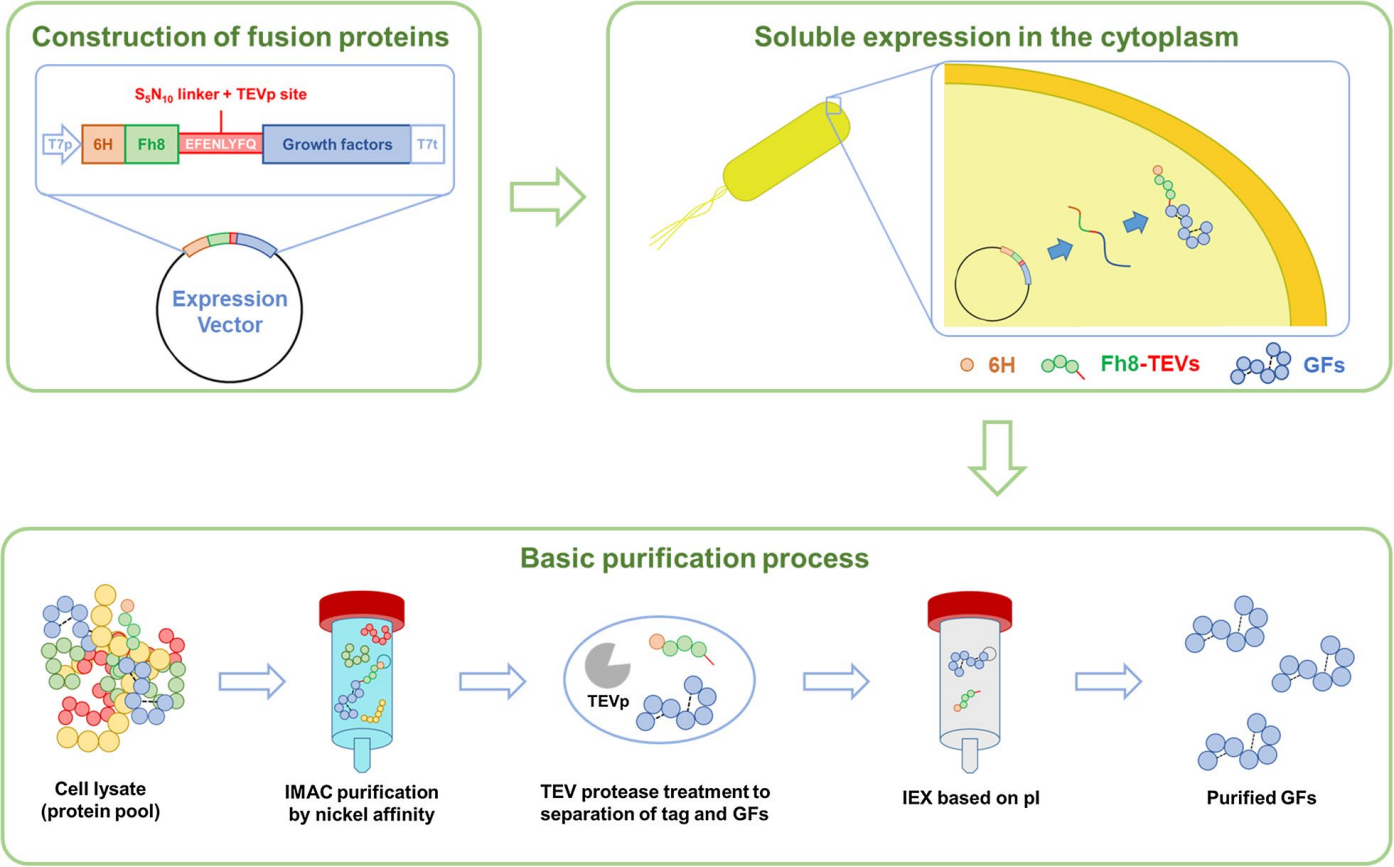
Schematic illustration of the GF production approach. GFs were fused with 6HFh8 via S_5_N_10_ linker sequence and TEV protease cleavage site. The fusion proteins were expressed in soluble form in the cytoplasm. They were first purified by HisTrap chromatography, and the target proteins were obtained by TEV protease cleavage. The proteins were then purified by IEX (HiTrap CM or HiTrap SP) chromatography based on the pI value. Dashed lines (bottom panel) indicate disulfide bonds

In the current study, we aimed to develop an industrially viable bacterial expression system using 6HFh8 as a fusion partner that enables production of various human GFs, rendering high yield and purity. We fused 6HFh8 via an S_5_N_10_ linker and tobacco etch virus (TEV) protease cleavage site to the N-terminus of GFs in the P1′ position (X) of TEV protease cleavage site (ENLYFQ/X), for the production of the authentic GF (Fig. 1). In addition, for mass production, we developed fed-batch fermentation and purification approaches using aFGF and VEGF165 as representative proteins that lack disulfide bonds and as representative cysteine-rich proteins, respectively. Using the devised approach, we successfully obtained large quantities of highly pure GFs with proliferative activity that was similar to that of their commercial equivalents. We conclude that this approach can be used for large-scale production of mammalian proteins in *E. coli*.

## Results

### Construction of an expression vector for human GFs fused to Fh8

To enhance the soluble expression of human GFs in *E. coli*, Fh8 was used as a solubility-enhancing fusion partner. It was fused at the N-terminus of GFs via a stable linker peptide S_5_N_10_ (Fig. 1) [36]. Further, hexa-histidine (6H) was attached to the N-terminus of the Fh8 tag (6HFh8) to facilitate the purification of fusion proteins. A TEV protease recognition site (ENLYFQ-G/S) was inserted between the S_5_N_10_ linker and the N-terminus of GF to allow cleavage of the fusion protein into the comprising proteins. To obtain authentic GFs from the fusion proteins (6HFh8-GFs), glycine or serine at the seventh position (P1′ position) of the TEV protease recognition sequence was replaced by the first amino acid of each GF. The resultant expression vectors were used to transform *E. coli* BL21 (*DE3*). The following human GFs were tested in the current study: acidic and basic FGFs (aFGF and bFGF, respectively), EGF, human growth hormone (hGH), IGF-1, VEGF165, KGF-1, placental GF (PGF), stem cell factor (SCF), and tissue inhibitor of metalloproteinase 1 (TIMP-1) (Additional file 1: Table S1).

### Production of human GFs fused to Fh8 in *E. coli* in shake-flask cultures

**Fig. 2:**
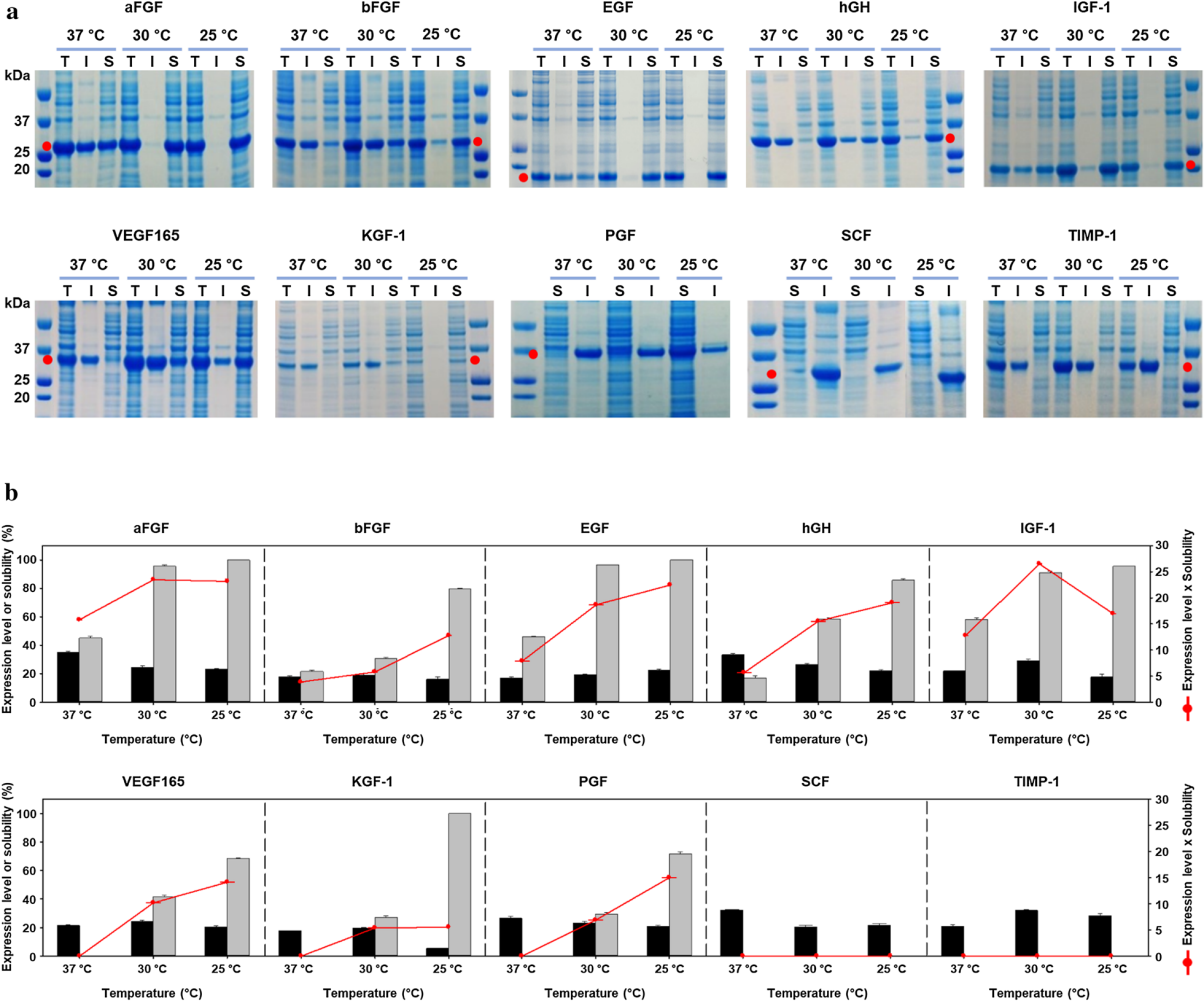
Expression of 6HFh8-GFs in flask culture. The proteins were induced by lactose in auto-induction media. They were then purified by HisTrap chromatography. **a** SDS-PAGE analysis of protein expression. Red dot, the fusion protein. I: insoluble fraction; S: soluble faction; T: total fraction. The images are representative of two independent experiments. **b** Quantitative analysis of protein expression. The bar graphs show percent expression (black bar) and solubility (gray bar) (primary y-axis), and the red line indicates expression level times solubility (secondary y-axis). Red line peak indicates the optimal temperature for fusion protein expression. Except for SCF and TIMP-1, all proteins were successfully expressed in soluble form at low temperature (25 °C and/or 30 °C). The expression level and solubility were analyzed by densitometry using ImageJ with two independent experiments

**Fig. 3:**
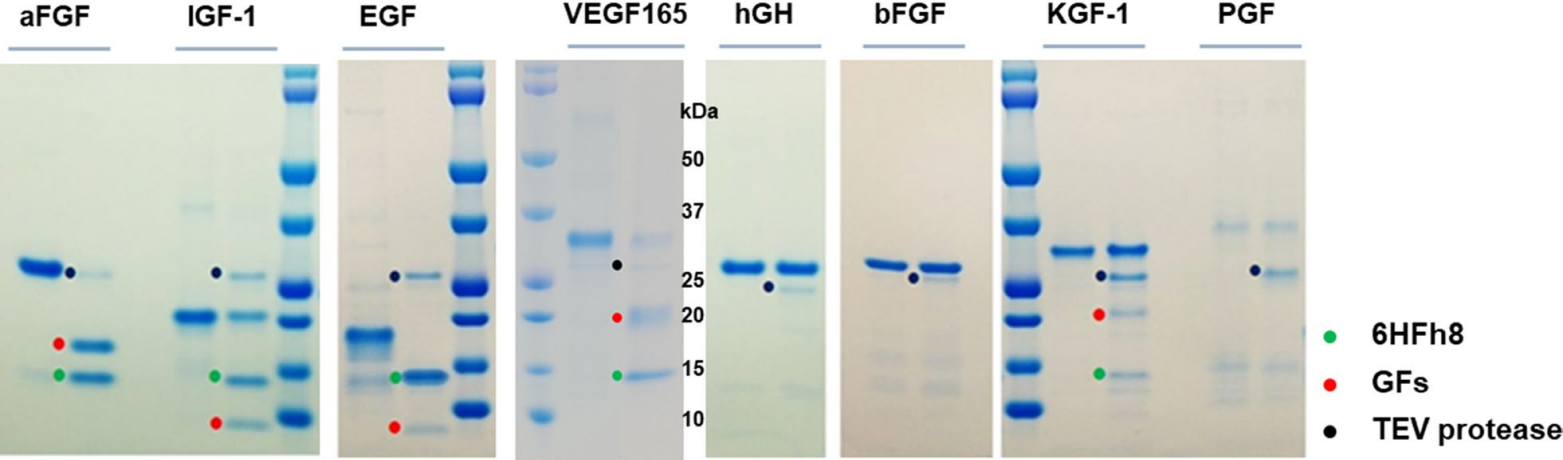
Analysis of the feasibility of original target protein liberation by TEV protease treatment. **a** The fusion proteins were purified by HisTrap chromatography and treated with TEV protease and the reaction mixtures resolved by SDS-PAGE. The images are representative of two independent experiments. C: after cleavage; F: fusion protein before cleavage

To determine the optimized expression condition for 6HFh8-GFs, recombinant *E. coli* cells transformed with each expression vector were cultured in shake flasks at three different temperatures (25 °C, 30 °C, and 37 °C). As shown in Fig. 2a, 6HFh8-aFGF, 6HFh8-bFGF, 6HFh8-EGF, 6HFh8-hGH, and 6HFh8-IGF-1 were expressed in soluble form at the three temperatures, while 6HFh8-VEGF165, 6HFh8-KGF-1, and 6HFh8-PGF were expressed in soluble form only at 25 °C and 30 °C. Unexpectedly, 6HFh8-SCF and 6HFh8-TIMP-1 were expressed only in insoluble form at the three temperatures. Considering cell growth and protein expression levels, the optimal temperature was 30 °C for 6HFh8-aFGF and 6HFh8-IGF-1; and 25 °C for 6HFh8-bFGF, 6HFh8-EGF, 6HFh8-hGH, 6HFh8-VEGF165, 6HFh8-KGF-1, and 6HFh8-PGF (Fig. 2b). To analyze whether GFs could be cleaved from the fusion proteins, soluble expressed fusion proteins were treated with TEV protease after primary purification by HisTrap chromatography. As shown in Fig. 3a, aFGF, IGF-1, EGF, and VEGF165, but not other GFs, were cleaved from the fusion proteins. N-terminal sequence analysis confirmed that the obtained aFGF, IGF-1, EGF, and VEGF165 were the authentic GFs (Table 1). The X in P6′ of EGF and IGF-1 indicated C because of the characteristics of the Edman sequence method. Additionally, we added the G in the P1′ position (between the TEV protease cleavage site Q and N-terminus of the GF) of the bFGF fusion protein to facilitate cleavage reaction (Additional file 2: Fig. S1). Consequently, the fusion protein was cleaved, although additional residue existed in the N-terminus of the target protein.

**Table 1:** N-terminal sequence results of aFGF, EGF, IGF, and VEGF165. Expected sequence indicates the reference sequence, and observed sequence indicates the result of the N-terminal sequence. C was detected as X due to the characteristics of the Edman sequence method

Protein	Sequence	Position									
		P1′	P2′	P3′	P4′	P5′	P6′	P7′	P8′	P9′	P10′
aFGF	Expected	F	N	L	P	P	G	N	Y	K	K
	Observed	F	N	L	P	P	G	N	Y	K	K
EGF	Expected	N	S	D	S	E	C	P	L	S	H
	Observed	N	S	D	S	E	X	P	L	S	H
IGF-1	Expected	G	P	E	T	L	C	G	A	E	L
	Observed	G	P	E	T	L	X	G	A	E	L
VEGF165	Expected	A	P	M	A	E	G	G	G	Q	N
	Observed	A	P	M	A	E	G	G	G	Q	N

### Production of 6HFh8-aFGF and 6HFh8-VEGF165 in 5-l fed-batch cultures

**Fig. 4:**
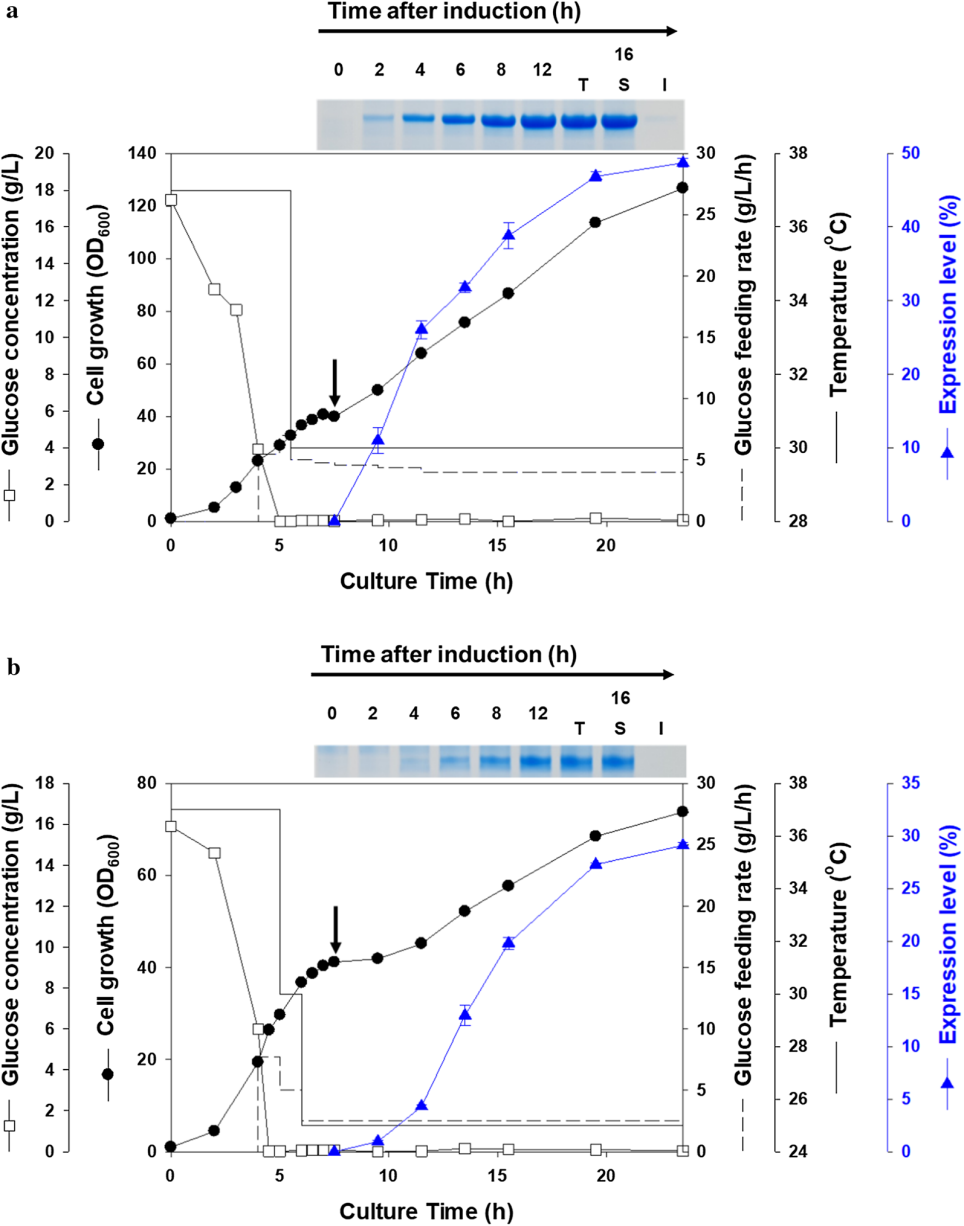
Protein expression in 5-l fermentation. Expression profiles of 6HFh8-aFGF (**a**) and 6HFh8-VEGF165 (**b**) are shown. SDS-PAGE images in the insets indicate the expression levels of fusion proteins at the indicated time after induction. Black arrows indicate the induction time. Values are averages from two independent experiments; errors are standard deviation

The complexity and number of intramolecular disulfide bonds in a protein can affect protein production in *E. coli* [25, 27]. The numbers of cysteine and intramolecular disulfide bonds in GFs analyzed in the current study are shown in Additional file 1: Table S1. For instance, aFGF has three cysteines but no intramolecular disulfide bonds, while VEGF165 has 16 cysteines and three intra- and two intermolecular disulfide bonds. Therefore, we then devised fermentation and purification approaches for aFGF and VEGF165 for their mass production in a reactor with a 2-l working volume. Glucose-limited fed-batch cultivation was used to improve the volumetric yield of *E. coli* harboring each expression vector (Fig. 4, Table 2). After complete consumption of the original glucose content 5 h after inoculation, additional glucose was fed into the fermenter at a rate of 6 g/l/h of glucose. Cell density approximately reached 35 OD_600_ units after 1 h of glucose feeding; the culture temperature was then decreased to 30 °C and 25 °C for the soluble expression of 6HFh8-aFGF and 6HFh8-VEGF165, respectively. Lactose, as an expression inducer, was added to a final concentration of 15 g/l after 1.5-h cultivation, and the fermentation was continued for a total fermentation time of 23.5 h.

**Table 2:** Production of 6HFh8-fused aFGF and VEGF165 using fed-batch fermentation

GF	End-point OD_600_	Dry cell weight (g/l) (wet cell) (g)	Expression level (%)^a^	Fusion protein (tag-free protein) (g/l)	Solubility (%)
aFGF	126.8 ± 0.0	44.4 (254.6)	48.8 ± 0.6	9.7 (5.1)	98.2
VEGF165	73.8 ± 0.1	25.8 (150.2)	29.1 ± 0.3	3.4 (1.6)	100

The data are shown as mean ± standard deviation from duplicate experiments

^a^Expression level and solubility were analyzed by densitometry using ImageJ with duplicate experiments

As shown in Fig. 4a, 6HFh8-aFGF was expressed 2 h after the induction, and its presence in the soluble fraction continually increased. Ultimately, the expression level of 6HFh8-aFGF reached 48.8% at the end of the experiment (final solubility 98.2%). The final OD_600_ reached 126.8, which was equivalent to 254.6 g of wet cell weight (calculated as 44.4 g of dry cell weight per liter), with a 6HFh8-aFGF yield of approximately 9.7 g/l. As shown in Fig. 4b, 6HFh8-VEGF165 was expressed 4 h after lactose induction, with its presence in the soluble fraction also increasing. Overall, the expression level of 6HFh8-VEGF165 reached 29.1% at the end of the experiment (final solubility 100%). The final culture OD_600_ reached 73.8, equivalent to 150.2 g of wet cell weight (calculated as 25.8 g of dry cell weight per liter), with the 6HFh8-VEGF165 yield of approximately 3.4 g/l. These observations demonstrated the feasibility of using the devised expression strategy for GF mass production in a soluble form.

### Development of a purification approach for aFGF

**Fig. 5:**
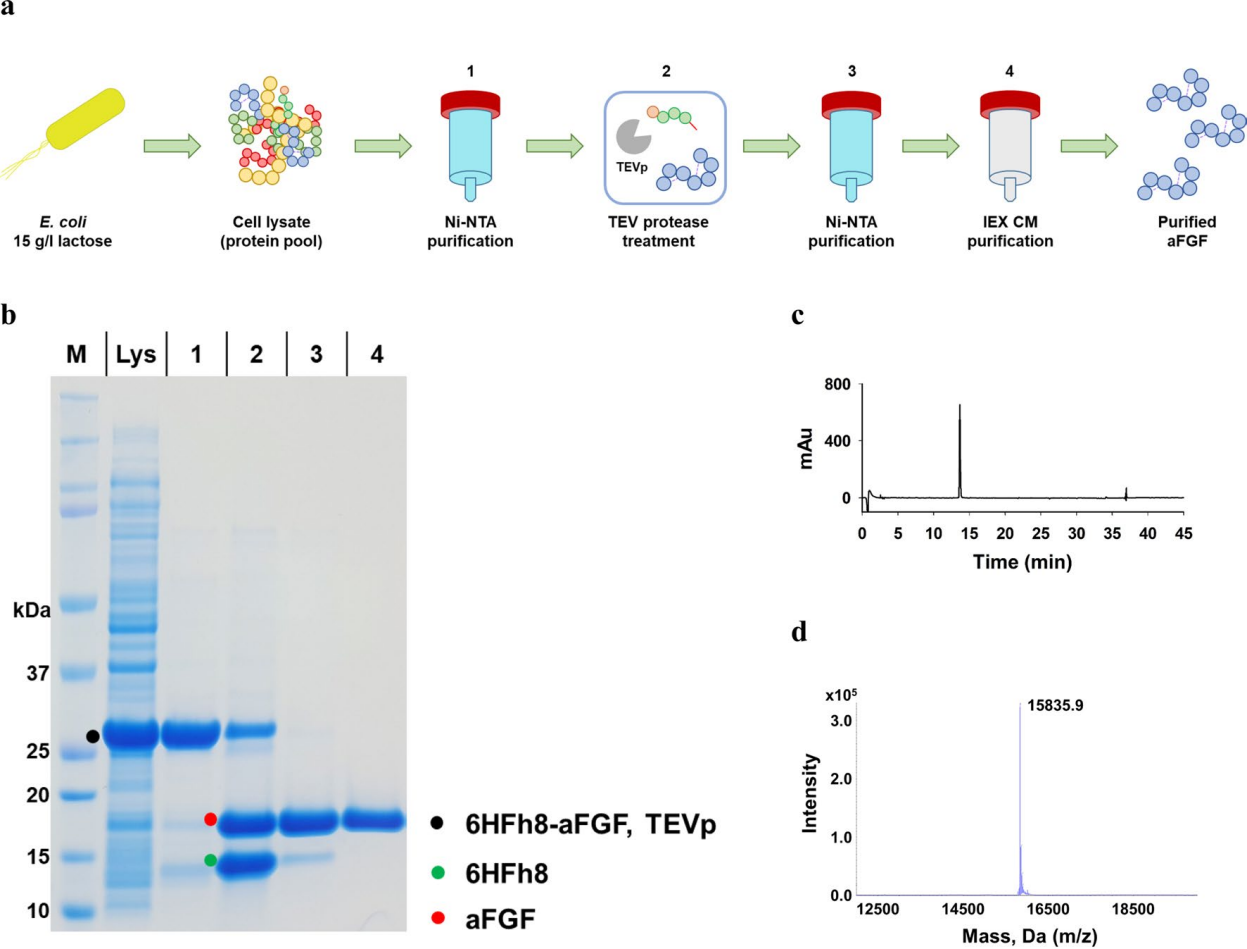
Purification and analysis of aFGF. **a** The overall purification steps. **b** SDS-PAGE analysis of samples from each purification step. **c** C18 RP-HPLC trace. **d** LC–MS (Q-TOF) analysis. M: marker; Lys: supernatant after sonication; 1: HisTrap purification; 2: after TEV protease treatment (the band at 28 kDa is TEVp); 3: HisTrap purification; 4: HiTrap CM purification (final product). The data are representative of three replicated experiments

To obtain a highly purified aFGF and VEGF165, we developed a fusion protein-specific purification process for each of the GF. As shown in Fig. 5a, the purification process for aFGF consisted HisTrap chromatography, TEV protease treatment, HisTrap chromatography for the removal of Fh8 and TEV protease, and cation-exchange chromatography. The cell lysate supernatant (Fig. 5b, lane Lys) was loaded onto HisTrap chromatography column and eluted with 500 mM imidazole. A prominent protein band of approximately 26.7 kDa, corresponding to 6HFh8-aFGF, was observed (Fig. 5b, lane 1). To increase of cleavage efficacy, the additives Triton X-100 and β-mercaptoethanol were used as additives (Additional file 3: Fig. S2a). β-mercaptoethanol showed better effect on the decrease of aggregation and the increase of cleavage efficiency than Triton X-100. Thus, the target protein was cleaved from the fusion protein by TEV protease in the presence of β-mercaptoethanol, overnight at 4 °C, with dialysis (Fig. 5b, lane 2) [37]. The aFGF and a small amount of the fusion partner (6HFh8 with the S_5_N_10_ linker) were eluted in an early elution fraction (50 mM imidazole) (Fig. 5b, lane 3), and TEV protease and most remaining fusion partners were eluted in a late elution fraction (> 250 mM imidazole) from the HisTrap column. Finally, highly purified aFGF was obtained by HiTrap CM chromatography after elution with 250 mM NaCl and removal of the remaining impurities (Fig. 5b, lane 4). The purity of the resultant aFGF exceeded 99%, as determined by C18 reversed-phase high-performance liquid chromatography **(**RP-HPLC) (Fig. 5c). Liquid chromatography-tandem mass spectrometry (LC–MS/MS) analysis indicated that the *m/z* of purified aFGF was 15,836, which was the same as the theoretical value (15,836); aFGF was hence detected with a 0% error (Fig. 5d). The yield from each purification step is summarized in Table 3. Approximately 59.6 mg of purified aFGF was obtained from 81.0 mg of aFGF in the crude extract (1.57 g of wet cells), with a purification yield of 73.5%. In other words, 4.8 g/l of tag-free aFGF (theoretical yield, 5.1 g/l, as calculated from the dry cell weight based on the ratio of the target protein to fusion tag in the fusion protein) was obtained from 5-l fermentation.

**Table 3:** Purification of aFGF from 6HFh8-aFGF fusion protein expressed in *E. coli*

Purification step	Concentration (mg/ml)^a^	Volume (ml)	Total protein (mg)	Fusion protein (and tag-free) (mg)	Purity (%)^a^	Yield (%)^b^
Crude extract	4.6 ± 0.1	70.0	322.7 ± 8.9	153.0 ± 2.8 (81.0 ± 1.5)	47.4	100.0
HisTrap, 5 ml	2.5 ± 0.0	67.3	165.6 ± 2.9	142.5 ± 2.5 (75.5 ± 1.3)	86.1	93.2
HisTrap, 5 ml	1.6 ± 0.0	50.1	77.9 ± 2.5	NA (72.3 ± 2.3)	92.8	89.2
HiTrap CM, 5 ml	0.7 ± 0.0	80.5	59.6 ± 2.3	NA (59.6 ± 2.3)	100.0	73.5

The data are shown as mean ± standard deviation from triplicate independent experiments

^a^Purity was analyzed by densitometry using ImageJ and C18 RP HPLC

^b^Yield was calculated by dividing the tag-free protein of each purification product by tag-free protein in crude extract

### Development of a purification approach for VEGF165

**Fig. 6:**
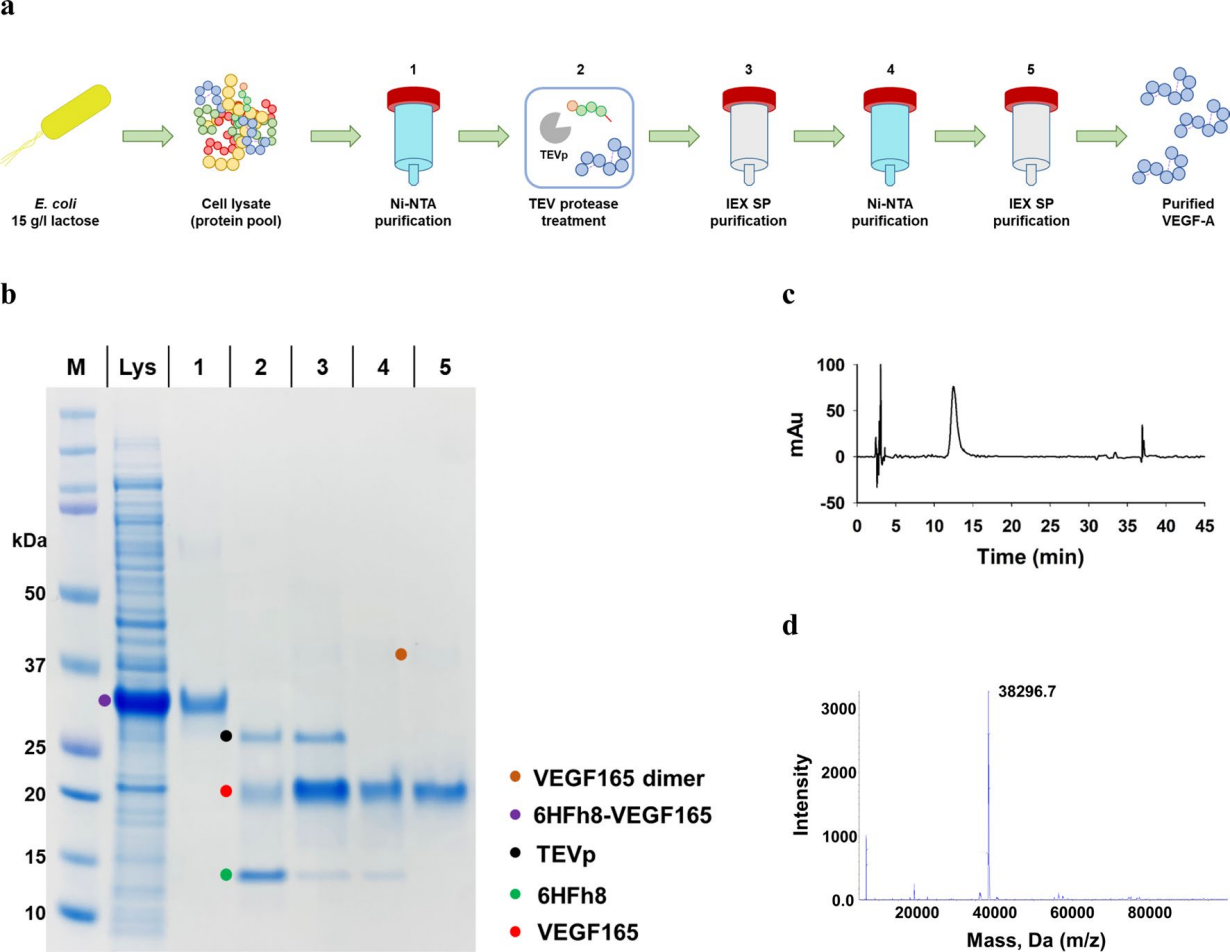
Purification and analysis of VEGF165. **a** The overall purification steps. **b** SDS-PAGE analysis of samples from each purification step. **c** C18 RP-HPLC trace. **d** LC–MS (Q-TOF) analysis. M: marker; Lys: supernatant after sonication; 1: HisTrap purification; 2: after TEV protease treatment (the band at 28 kDa is TEVp); 3: HiTrap SP purification; 4: HisTrap purification; 5: HiTrap SP purification (final product). The data are representative of three replicated experiments

The developed purification approach for VEGF165 is shown in Fig. 6a. It involved the following stages: HisTrap chromatography, TEV protease treatment, cation-exchange chromatography for tag removal, HisTrap chromatography for TEV protease removal, and a final cation-exchange chromatography step. The cell lysate supernatant (Fig. 6b, lane Lys) was loaded onto a HisTrap chromatography column and eluted with 500 mM imidazole. A prominent protein band of approximately 30.1 kDa corresponding to 6HFh8-VEGF165 was observed (Fig. 6b, lane 1). Prior to the TEV protease treatment, the eluted VEGF165 was dialyzed overnight at 4 °C, to remove NaCl and imidazole. As observed in aFGF, cleavage efficiency of VEGF165 fusion protein was enhanced by the addition of β-mercaptoethanol (Additional file 3: Fig. S2b). Thus, the target protein was cleaved from 6HFh8-VEGF165 by TEV protease in the presence of β-mercaptoethanol, overnight at 4 °C (Fig. 6b, lane 2) [37]. VEGF165, TEV protease, and a small amount of the fusion partner (6HFh8 with the S_5_N_10_ linker) were eluted in the early elution fraction (300 mM NaCl) (Fig. 6b, lane 3), and most fusion partner was removed in the unbound fraction by HiTrap SP chromatography. TEV protease was removed based on the affinity difference of VEGF165 and TEV protease to the HisTrap resin. Specifically, the dialyzed sample was loaded on the HisTrap column, and VEGF165 was eluted in 150 mM imidazole (Fig. 6b, lane 4). Finally, highly pure VEGF165 was obtained by HiTrap SP chromatography by eluting in 500 mM NaCl after removal of any remaining impurities (Fig. 6b, lane 5). The purity of the obtained VEGF165 exceeded 99%, as determined by C18 RP-HPLC (Fig. 6c). LC–MS/MS analysis indicated that the m/*z* of purified VEGF165 was 38,297, which was expected for a dimer (homodimer) of VEGF165 monomers (19,166) (Fig. 6d). The theoretical dimer size of VEGF165 is 38,332, and thus, dimeric VEGF165 was detected with a 0.09% error. The overview and yield of each purification step are presented in Table 4. Approximately 0.2 mg of purified VEGF165 was obtained from 16.5 mg of VEGF165 in the crude extract (0.9 g of wet cells), with a purification yield of 0.9%. Hence, 18.2 mg/l of tag-free VEGF165 (theoretical yield; 1.6 g/l, as calculated from the dry cell weight based on the ratio of the target protein to fusion tag in the fusion protein) was obtained from 5-l fermentation.

**Table 4:** Purification of VEGF165 from 6HFh8-VEGF165 fusion protein expressed in *E. coli*

Purification step	Concentration (mg/ml)	Volume (ml)	Total protein (mg)	Fusion protein (tag-free protein) (mg)	Purity (%)^a^	Yield (%)^b^
Crude extract	1.5 ± 0.0	100	147.5 ± 0.7	40.8 ± 0.2 (18.8 ± 0.1)	27.1	100
HisTrap, 5 ml	0.3 ± 0.0	88	30.4 ± 0.6	29.0 ± 0.6 (13.4 ± 0.3)	95.9	79.8
HiTrap SP, 5 ml	0.11 ± 0.0	11	1.3 ± 0.0	NA (2.9 ± 0.1)	80.4	6.1
HisTrap, 5 ml	0.04 ± 0.0	21	0.8 ± 0.0	NA (2.8 ± 0.1)	95.8	4.6
HiTrap SP, 5 ml	0.03 ± 0.0	6	0.2 ± 0.0	NA (0.2 ± 0.0)	> 99	0.9

The data are shown as mean ± standard deviation from triplicate independent experiments

^a^Purity was analyzed by densitometry using ImageJ and C18 RP HPLC

^b^Yield was calculated by dividing the tag-free protein of each purification product by tag-free protein in crude extract

### Analysis of the properties of purified aFGF and VEGF165

**Fig. 7:**
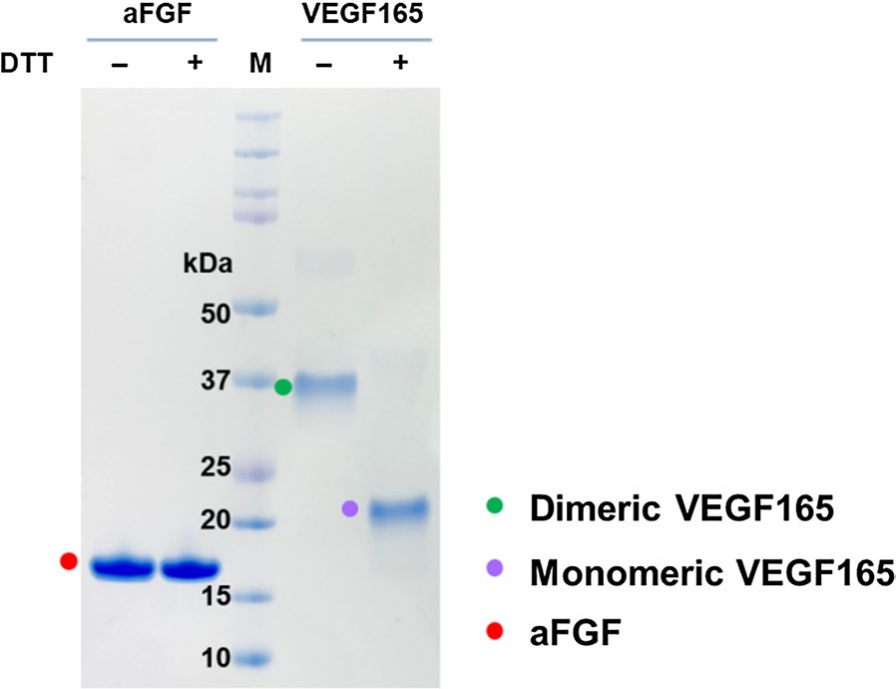
Disulfide bond formation by purified GFs, and structural properties of aFGF and VEGF165. Disulfide bond formation was analyzed indirectly based on the protein size difference after DTT treatment (100 °C, 5 min). The data are representative of three replicated experiments

While aFGF forms monomers (15.8 kDa), VEGF165 forms homodimers (38.4 kDa). That is because aFGF lacks intermolecular disulfide bonds and VEGF165 has two intermolecular disulfide bonds (Additional file 1: Table S1). VEGF165 forms a homodimer if it is correctly folded and active [17, 38, 39]. To verify the conformations of aFGF and VEGF165, molecular masses of the purified proteins in the absence and presence of the reducing agent dithiothreitol (DTT) were compared. As anticipated, the observed molecular mass of purified aFGF was unaffected by the DTT treatment (Fig. 7). However, the observed molecular mass of VEGF165 was reduced from almost 40 kDa under non-reducing conditions to 20 kDa under reducing conditions. This confirmed the native folds of the two purified proteins.

**Fig. 8:**
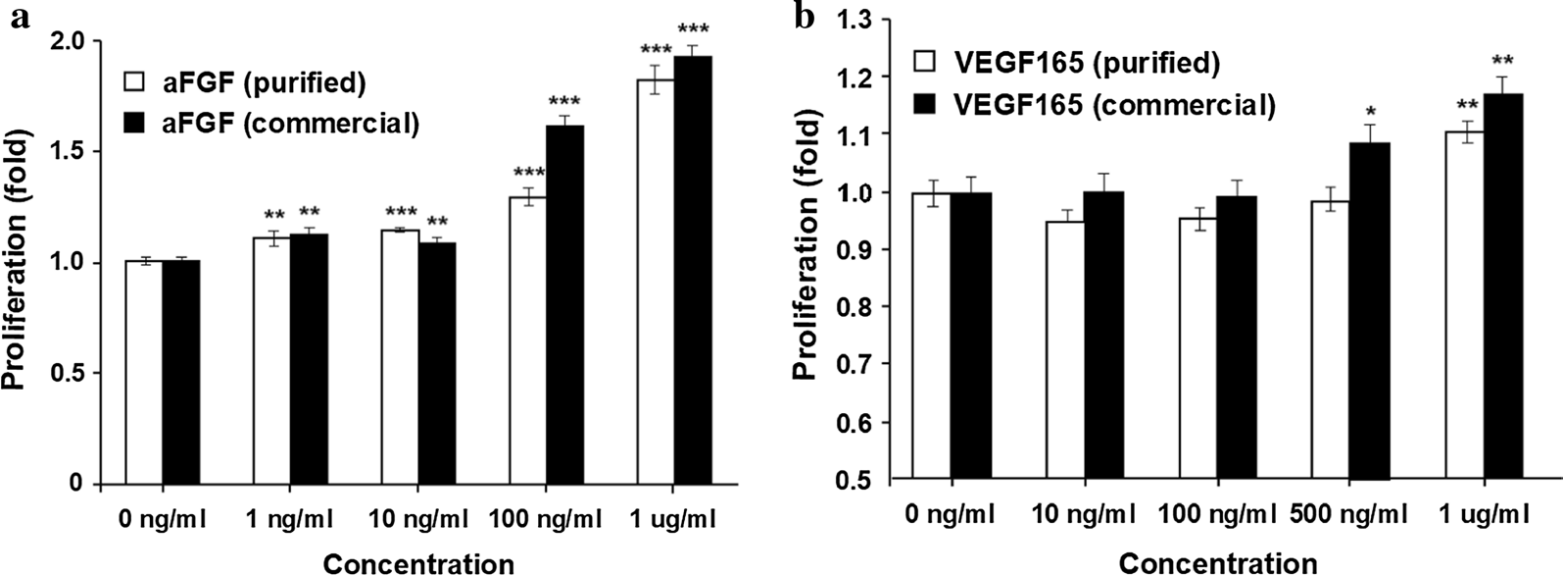
The proliferative effect of recombinant GFs. **a** The proliferative effect of aFGF on HDF cells. **b** The proliferative effect of VEGF165 on HUVECs. The data are shown as mean ± standard deviation from three replicated experiments. The value of *p* was calculated by Student’s *t*-test for comparison with the untreated group. *p ≤ 0.05, **p ≤ 0.01, and ***p ≤ 0.001

To analyze the biological activities of the purified aFGF and VEGF165 proteins, we investigated their proliferative activities in vitro. The MTT assay was used to evaluate the effect of aFGF on human dermal fibroblasts (HDF; a human skin cell) and the effect of VEGF165 on human umbilical vein endothelial cells (HUVECs) (Fig. 8). To verify the dose-dependent effect of purified proteins, the HDF and HUVEC cultures were treated with different concentrations (from 0 to 1 μg/l ) of these proteins. The purified and commercial aFGF proteins had a similar proliferative effect at 100 ng/ml to 1 μg/ml (181% and 191% cell proliferation by the purified and commercial aFGF, respectively, at 1 μg/ml compared with that of the untreated group), with no effect at low concentrations (0 to 10 ng/ml) (Fig. 8a). The purified and commercial VEGF165 proteins also had a similar proliferative effect, with cell proliferation significantly induced by 500 ng/ml to 1 μg/ml of these proteins (122% and 135% cell proliferation by the purified and commercial VEGF165, respectively, at 1 μg/ml compared with that of the untreated group), and no effect at low concentrations (0 to 100 ng/ml) (Fig. 8b). This demonstrated the activity of GFs purified by the novel approach.

## Discussion

Improvement of soluble expression of proteins is important for increasing the production yield of protein. It is especially important when a mammalian protein of interest is produced in microorganisms, such as bacteria. GFs are a protein type produced in a heterologous host [1, 15–20]. To meet increasing demand, highly productive systems, such as *E. coli* or yeast, are used for GF production. In general, GFs are small (6.3 to 23 kDa) and cysteine-rich, with intermolecular disulfide bonds (Additional file 1: Table S1). We here presented a novel and efficient approach for soluble overproduction of GFs in the cytoplasmic fraction of *E. coli*, accomplished by N-terminal fusion with the 6HFh8 protein as a solubility-enhancing fusion partner, a linker, and a TEV protease cleavage site. Eight of the ten GFs were overexpressed and authentic aFGF, IGF-1, EGF, and VEGF165 were cleaved from the fusion proteins by TEV protease. To verify the GF production strategy, we developed the fermentation and purification approaches for the aFGF and VEGF 165 that were highly purified and active. To our knowledge, this is the first report of the expression and production of various GFs in *E. coli* using a single tag. This approach provides an effective strategy for producing mammalian protein in *E. coli*, on an industrial scale.

We obtained 0.2 mg purified VEGF165 per 0.9 g of wet cells, with 0.9% purification yield (Table 4). The theoretical production yield of VEGF165 from 5-l fermentation was 1.6 g/l and the production yield was calculated as 18.2 mg/l. A number of possible reasons exist for the difference in VEGF165 yield (1.6 g/l vs. 18.2 mg/l), such as, loss during purification (especially during affinity chromatography and during dialysis after the first HisTrap purification) and during the TEV protease treatment. Furthermore, tag-free VEGF165 was also bound to the HisTrap column. This made it difficult to separate VEGF165 from the overall protein pool. Therefore, during the development of the VEGF165 purification approach, we changed the HisTrap chromatography step (the second step) to HiTrap SP FF 5 ml chromatography (Fig. 5). When the yield of each purification step was calculated by the same method used with purification of aFGF, the second purification yield was 7.6%, the third purification yield was 75.2%, and the fourth purification yield was 20.7%. A slight decrease in the purification yield in the second and forth purification steps (both HiTrap SP chromatography) indicated that the dramatic change in buffer pH and buffer compound NaCl affected the purification yield because of the aggregation of protein. Furthermore, a greater decrease in purification yield in the second purification step was caused by the aggregation during TEV protease treatment. Hence, the optimization of TEV protease treatment conditions, dialysis, and the second and forth purification steps can drastically increase VEGF165 production yield (Table 4). The volumetric production yield of 18.2 mg/l was 11 times higher than that previously reported (1.66 mg/l) by Nguyen et al. [17] using the MBP tag, although the purification yield in the current study was lower than that reported in the previous study (2.9%). In the case of aFGF, it is easily produced (via one-step purification) without any tag in *E. coli* mutant strain SHuffle T7 with a very high production yield (1.500 g/l) and 94% purity with 70% purification yield; however, thus purified aFGF contains N-terminal methionine [19]. In the current study, the volumetric production yield of the devised approach was 4.8 g/l, with nearly 100% pure protein. As another benefit of using the devised approach, the purified aFGF can be easily used as a cosmeceutical or medical additive without further toxicity testing because it lacks additional (non-innate) residues.

There are two important points to consider when cleaving the GF from the fusion protein, i.e., production of the target protein without additional residues and aggregation of the target protein after removal of the fusion partner. In the current study, we addressed the first point by designing the TEV protease cleavage site. The cleavage site of TEV protease is ENLYFQ/X, where X is G or S, although some researchers have reported that the two residues, G and S, can be replaced with A, M, N, H, Y, Q, and F [40, 41]. In the current study, we fused GFs with an Fh8 tag via the TEV protease cleavage site ENLYFQ to subsequently obtain the authentic GFs. Therefore, X was replaced with the N-terminal residue of the target GF, namely, G (IGF-1), A (VEGF165), F (aFGF and hGH), P (bFGF), N (EGF), C (KGF-1 and TIMP-1), L (PGF), and E (SCF). As shown in Fig. 3, the aFGF (F), IGF-1 (G), EGF (N), and VEGF165 (A) constructs were cleaved, while the hGH (F), bFGF (P), KGF-1 (C), and PGF (L) constructs were not. L was not a candidate replacement amino acid, and hence, the PGF fusion protein was not cleaved. Further, bFGF fusion was not cleaved because the R-group of P forms a unique folding structure that poses a steric hindrance for the protease [40, 42]. hGH and KGF-1 were also not cleaved. The N-terminal sequence of hGH is FP; hence, the TEV protease cleavage was inhibited by P in the second position [42, 43]. The N-terminus sequence of KGF-1 is CNDMTP; P is the sixth amino acid and, hence, possibly too far to affect the cleavage. However, only a small portion of the fusion protein was cleaved. Surprisingly, the N-termini of IGF-1 and VEGF165, GP and AP, respectively, were cleaved with by the TEV protease, but not a perfectly due to the P or structural properties of multiple disulfide bonds. Hence, it seems to be that the cleavage efficiency was influenced on the sequence of P1′ and P2′ and number of disulfide bonds. Additionally, we anticipated that the addition of G or A between the TEV protease cleavage site (terminal Q) and the GF N-terminus would improve the cleavage efficacy. The addition of G indeed allowed the cleavage of Fh8 and bFGF from the fusion protein (Additional file 1: Fig. S1). Hence, we suggest that the addition of G would further improve the cleavage efficacy.

As mentioned above, the second key point is the aggregation of target proteins, often observed after the removal of the fusion partner [44]. In the current study, during the purification of aFGF and VEGF165, several impure protein aggregates were formed after TEV protease treatment, and the portion of tag-free aFGF and VEGF165 precipitated together (Additional file 3: Fig. S2a and b). We therefore optimized the TEV protease treatment conditions by using additives such as β-mercaptoethanol, Tween-20, and Triton X-100 [16, 36, 45–48]. In the case of aFGF, almost half the aFGF and fused aFGF aggregated after treatment with 5% (w/w) TEV protease in 1× phosphate-buffered saline (PBS) supplemented with 150 mM NaCl (Additional file 3: Fig. S2a). The addition of 1% (v/v) Triton X-100 reduced the amount of aggregated protein by approximately one-half compared with a reaction without Triton X-100. Further, the addition of both 10 mM β-mercaptoethanol and 1% (v/v) Triton X-100 considerably reduced protein aggregation. Moreover, as shown in Fig. 5b, lane 2, the presence of 10 mM β-mercaptoethanol prevented the aggregation of aFGF with a similar effect as that of treatment with 10 mM β-mercaptoethanol and 1% (v/v) Triton X-100 together (predicted by the similar intensity of aFGF and 6HFh8 bands). In case of VEGF165, similar to aFGF, almost half the fused VEGF165, VEGF165, and TEV protease aggregated using 10% (w/w) TEV protease (the difference between S + I and S is I; Additional file 3: Fig. S2b). However, unlike with aFGF, the addition of 1% (v/v) Triton X-100 had no discernible effect on protein aggregation, and a large portion of the fused VEGF165 remained uncleaved. Moreover, when both 10 mM β-mercaptoethanol and 1% (v/v) Triton X-100 were present, almost all fused VEGF165 was cleaved, and tag-free VEGF165 was observed in the supernatant. Further, the optimal conditions of TEV protease treatment of aFGF and VEGF165 (with 10 mM β-mercaptoethanol) did not affect protein activity (Fig. 8). Therefore, we predict that the addition of β-mercaptoethanol in the dialysis buffer can reduce the aggregation during the purification of VEGF165 for increase the purification yield. Hence, the novel strategy for the production of GFs with a 6HFh8 fusion tag in *E. coli* can be used for protein production on an industrial scale by optimizing the purification process.

## Conclusions

We here constructed GFs fused to 6HFh8, a linker, and a TEV protease cleavage site to facilitate their mass production in *E. coli*. Among the fusion proteins, representative proteins aFGF, lacking disulfide bonds, and VEGF165, a cysteine-rich protein, were successfully expressed in 5-l fed-batch fermentation, indicating the possibility of mass production. Both GFs were produced with high purity and activity, demonstrating a dramatic improvement in the production yield. These findings indicate that 6HFh8 can be used to produce human-derived proteins with multiple disulfide bonds, including GFs, in *E. coli* on an industrial scale.

## Materials and methods

### Construction of protein expression vectors and strains

Genes encoding the GFs with 6HFh8 were codon-optimized and synthesized by DNA 2.0 (ATUM, Menlo Park, CA, USA). The fusion proteins were constructed as following our previous report [36]. Briefly, hexa-histidine fused Fh8 (6HFh8) was fused to N-terminus of GFs via the S_5_N_10_ linker and TEV protease cleavage site (ENLYFQ-G/S) in the P1′ position. The genes were inserted into a pET-30a vector (Novagen, Madison, WI, USA) pre-digested with the restriction enzymes Nde I and Xho I using T4 DNA ligase (Takara Bio, Otsu, Japan). The resultant plasmids were heat-shocked to transform *E. coli* DH5α (RBC Bioscience, New Taipei City, Taiwan). For the expression of fusion proteins, the recombinant plasmids were used to transform *E. coli* BL21 (DE3) (NEB, Ipswich, MA, USA).

### Bacterial cultivation for protein production and analysis of protein expression

The flask culture medium and 5-l fed-batch fermentation medium in the current study were described in our previous experiments [36]. Briefly, flask cultivations were performed in an auto-induction medium [per liter: 0.5 g glucose, 3 g glycerol, 2 g lactose, 0.15 g MgSO_4_·7H_2_O, 10 g yeast extract, 16 g tryptone, 3.3 g (NH_4_)_2_SO_4_, 6.8 g KH_2_PO_4_, and 7.1 g Na_2_HPO_4_·12H_2_O, and 1 ml of trace element solution containing 0.5 g/l CoCl_2_·6H_2_O, 65 g/l FeSO_4_·7H_2_O, 3 g/l MnSO_4_·5H_2_O, 5 ml/l H_2_SO_4_, 0.08 g/l KI, 6 g/l CuSO_4_·5H_2_O, 20 g/l ZnCl_2_, 0.02 g/l H_3_BO_3_, 0.2 g/l Na_2_MoO_4_·2H_2_O, and 0.2 g/l biotin) at three different temperatures (25 °C, 30 °C, and 37 °C). Recombinant *E. coli* cells harboring the GF expression plasmids were cultured in 2 ml of Luria-Bertani (LB) medium supplemented with 50 μg/ml kanamycin at 37 °C overnight. Then, 0.5 ml of culture was transferred to 50 ml of the auto-induction medium supplemented with 50 μg/ml kanamycin in a 250-ml baffled flask and incubated for 12 h at 37 °C, or 24 h at 30 °C or 25 °C, at 200 rpm.

In another set of experiments, 5-l fed-batch fermentations were performed in the following initial medium: 15 g/l glucose, 1 g/l MgSO_4_·7H_2_O, 10 g/l yeast extract, 10 g/l casein peptone, 10 g/l (NH_4_)_2_SO_4_, 0.5 g/l NaCl, 3 g/l Na_2_HPO_4_·12H_2_O, 3 g/l KH_2_PO_4_, and 1 ml/l trace element solution as described above. The inoculum for bioreactor cultures was prepared as follows. For the primary seed culture, a single colony from LB agar supplemented with 50 μg/ml kanamycin was inoculated into 50 ml of LB medium with 50 μg/ml kanamycin and cultured overnight at 37 °C and 200 rpm. For the secondary seed culture, 2 ml of the primary seed culture was inoculated into 200 ml of LB medium supplemented with kanamycin and incubated at 37 °C and 200 rpm for 5 h. The 5-l fed-batch fermentation was performed in a 2-l initial working volume in a 5-l bioreactor. The culture conditions were controlled and maintained as follows: cell growth temperature, 37 °C; pH adjusted to 7.0 by the addition of ammonium hydroxide; dissolved oxygen, above 30%; airflow, 1 vvm; and automatic agitation controlled between 200 rpm and 900 rpm. All the controlled conditions were monitored, and glucose levels were analyzed by a glucose analyzer (YSI 2700 Biochemistry Analyzer; Yellow Springs Instrument Co., Yellow Springs, OH, USA). When the glucose present in the initial medium was entirely consumed, additional glucose was fed at an initial feeding rate of 8 g/l/h. After adjusting the temperature to 25 °C or 30 °C, the feeding rate of glucose was decreased to 6 g/l/h or 4 g/l/h, respectively. Further, 15 g/l lactose was added to promote the expression of the recombinant protein gene, and the incubation continued for a total culture time of 23.5 h.

To analyze the expression level and solubility, 1 ml of cells with OD_600_ was harvested by centrifuging at 15,814×*g* at 4 °C for 1 min, and remaining cells were harvested by centrifuging at 6520×*g* at 4 °C for 20 min for storage. After washing twice with PBS, the pellet was re-suspended in 1 ml PBS and disrupted by a sonicator (Cole-Parmer Instruments, Vernon Hills, IL, USA) at 40% amplitude, pulse 5 s on and 5 s off, for 10 min on ice. The debris was removed by centrifugation at 15,814×*g* at 4 °C for 20 min. Protein concentration was determined by Pierce™ BCA protein assay kit (Thermo Scientific, Waltham, MA, USA), and absorbance was measured at 550 nm by the plate reader Infinite 200 PRO (TECAN, Männedorf, Switzerland). Protein expression was evaluated by loading the protein onto 4–12% Bis–Tris Plus SDS-PAGE gel (Thermo Scientific) and running it at 170 V, 500 mA, for 35 min, followed by staining with InstantBlue (Abcam, Cambridge, UK).

### Purification of aFGF and VEGF165

All purification steps were performed using a ÄKTAprime plus chromatography system (GE Healthcare, Little Chalfont, UK), and the purification process was modified from that reported in previous studies [17, 19, 36]. In detail, the cells were resuspended in 50 ml or 70 ml of each HisTrap binding buffer (1× PBS with 150 mM NaCl or 50 mM Tris–HCl, pH 8.0, and 300 mM NaCl) and disrupted by sonication on ice at 40% amplitude, with pulse on for 5 s and pulse off for 5 s, for a total of 2 h. The sonicated samples were centrifuged at 14,810×*g* and 4 °C for 20 min and filtered through 0.45-μm filters to remove the debris.

The soluble fraction containing recombinant aFGF (from 1.57 g of wet cells) was loaded on a HisTrap HP 5-ml column (GE Healthcare) pre-equilibrated with HisTrap binding buffer (1× PBS with 150 mM NaCl) at a flow rate of 1 ml/min. The washing and elution steps were performed using HisTrap binding buffer supplemented with 25 mM and 500 mM imidazole, respectively, at a flow rate of 3 ml/min. The eluted fractions were pooled and conducted the cleavage reaction. The TEV protease and the β-mercaptoethanol were added in the pooled fraction with target protein to TEV protease ratio of 1:10 (w/w) and the final concentration of 10 mM, respectively, to cleave 6HFh8 and dialyzed against 1× PBS with 150 mM NaCl at 4 °C overnight. The dialyzed sample was loaded onto the same column at a flow rate of 1 ml/min. Fractions eluted with HisTrap binding buffer with 50 mM imidazole at a flow rate of 3 ml/min were pooled and mixed with an equal volume of 20 mM sodium phosphate buffer (pH 6.0) to prevent protein aggregation. They were then dialyzed against the binding buffer (20 mM sodium phosphate buffer; pH 6.0) at 4 °C overnight. The dialyzed samples were loaded onto a HiTrap CM FF 5-ml column (GE Healthcare) pre-equilibrated with the binding buffer at a flow rate of 1 ml/min. The column was then washed with binding buffer supplemented with 160 mM NaCl. Recombinant aFGF was eluted with binding buffer supplemented with 300 mM NaCl at a flow rate of 3 ml/min.

The soluble fraction containing recombinant VEGF165 (from 0.9 g of wet cells) was applied onto a HisTrap HP 5-ml column pre-equilibrated with HisTrap binding buffer (50 mM Tris–HCl, pH 8.0, and 300 mM NaCl) at a flow rate of 1 ml/min. The column was washed with binding buffer supplemented with 75 mM imidazole, and the fusion protein was eluted in binding buffer supplemented with 500 mM imidazole at a flow rate of 3 ml/min. The fusion protein was dialyzed against 20 mM sodium phosphate, pH 6.0, with 10 mM β-mercaptoethanol at 4 °C overnight to remove imidazole and NaCl and to prevent protein aggregation. The TEV protease and the β-mercaptoethanol were added in the dialyzed protein with target protein to TEV protease ratio of 1:10 (w/w) and 10 mM, respectively, to cleave 6HFh8 at 4 °C overnight. The cleaved sample was applied onto a HiTrap SP FF 5-mL column (GE Healthcare, pre-equilibrated with 20 mM sodium phosphate, pH 6.0, with 50 mM NaCl) at a flow rate of 1 ml/min. The bound protein was eluted in binding buffer supplemented with 300 mM NaCl at a flow rate of 3 ml/min and dialyzed against 20 mM sodium phosphate, pH 6.0, with 50 mM NaCl. The dialyzed sample was loaded onto a HisTrap HP 5-ml column (pre-equilibrated with 20 mM sodium phosphate, pH 6.0, with 50 mM NaCl) at a flow rate of 1 ml/min. The bound protein was eluted using binding buffer supplemented with 150 mM imidazole at a flow rate of 3 ml/min and applied onto the HiTrap SP FF 5-ml column (pre-equilibrated with 20 mM sodium phosphate, pH 6.0, with 50 mM NaCl) at a flow rate of 1 ml/min. Recombinant VEGF165 was eluted in binding buffer supplemented with 500 mM NaCl at a flow rate of 3 ml/min. The purified aFGF and VEGF165 proteins were stored at 4 °C until further analysis.

### Analysis of the purity of obtained aFGF and VEGF165 by HPLC

The purified recombinant aFGF and VEGF165 were analyzed by HPLC (1200 Series; Agilent Technologies, Santa Clara, CA, USA) with an UV detector at 214 nm. The C18 RP column (Zorbax Eclipse XDB, 80 Å C18, 4.6 × 150 mm, 5 μm; Agilent Technologies) connected to an HPLC system was maintained at 40 °C. The column was pre-equilibrated with buffer A (0.1% of trifluoroacetic acid in distilled water) and 5% (v/v) buffer B (0.1% of trifluoroacetic acid in acetonitrile). The flow rate was 0.5 ml/min; the sample volume was 20 μl, and the run time for each sample was 45 min.

### N-terminal sequencing and LC–MS/MS

Protein N-terminal sequences were obtained after transferring the purified recombinant aFGF and VEGF165 proteins to a polyvinylidene difluoride membrane using a Procise ABI 492 protein sequencer (Applied Biosystems, Foster City, CA, USA). The authenticity of purified proteins was verified by native mass spectrometry at eMASS (Seoul, Republic of Korea). Samples were analyzed following the service provider’s protocol. Briefly, they were first resolved by UHPLC Ultimate 3000 (Thermo Scientific) on an ACQUITY-C8 column (2.3 × 130 mm, 1.7 μm; Waters, Milford, MA, USA). Mobile phases A [H_2_O/formic acid, 100/0.2 (v/v)] and B [acetonitrile/formic acid, 100/0.2 (v/v)] were used for analysis. Approximately 10 μl of sample was injected for analysis and separated using a gradient of B in A from 5% to 100% for 12 min. Protein native mass was detected by using TripleTOF 5600 + (AB SCIEX, Framingham, MA, USA).

### Indirect analysis of disulfide bond formation by purified GFs

A 5× SDS-PAGE loading dye was prepared: 250 mM Tris–HCl, pH 6.8, 10% (w/v) SDS, 0.25% (v/v) bromophenol blue, and 50% (v/v) glycerol, with or without 100 mM DTT. The purified aFGF and VEGF proteins were then mixed with the loading dye and boiled at 100 °C for 5 min. The protein was loaded onto 4–12% Bis–Tris Plus SDS-PAGE gel and run at 170 V, 500 mA, for 35 min, followed by staining with InstantBlue.

### Proliferation assay with purified aFGF and VEGF165

The proliferative effect of purified aFGF and VEGF165 was investigated by the MTT assay using HDF (ATCC, Manassas, VA, USA) and HUVECs (ATCC), respectively. The cells were maintained in IMDM medium (Thermo Scientific) supplemented with 10% (v/v) fetal bovine serum at 37 °C with 5% CO_2_. The cells were seeded in a 96-well plate at a density of 1 × 10^4^ cells/well. After 24 h incubation, the spent medium was removed, and 100 μl of serum-free medium with purified aFGF or VEGF165 (0–1 μg/ml protein) and commercial aFGF (Merck, Darmstadt, Germany) or VEGF165 (Merck) were added, and incubated at 72 h. Following this, 10 μl of CCK-8 reagent (Dojindo Laboratories, Kumamoto, Japan) was added, and sample absorbance was measured at 450 nm by the plate reader Infinite 200 PRO after 2–3 h incubation at 37 °C.

### Statistical analysis

All data were obtained from the independent experiments are presented as the mean ± standard deviation. The data were analyzed with Student’s *t*-test. Analysis of variance (ANOVA) was performed for relevant data. Values of *p* ≤ 0.05 were considered statistically significant, and values of *p* ≤ 0.01 were considered highly significant.

## Supplementary information

**Additional file 1: Table S1.** Structural properties and molecular masses of growth factors overproduced in the current study. ‒, the range of specific region (heparin binding region in the structural properties) and protein without signal peptide and pro-region; ↔, the binding position of each residue.

**Additional file 2: Figure S1.** Enhancement of TEV protease cleavage efficiency by modification of the N-terminus of the protein of interest. Glycine was inserted between the TEV protease cleavage site and N-terminus of bFGF, the protein of interest. The fusion protein was purified by HisTrap chromatography and cleaved by the TEV protease. The protein mixtures were resolved on 4–12% Bis–Tris Plus SDS-PAGE gel. The image is representative of two independent experiments

**Additional file 3: Figure S2.** TEV protease treatment of aFGF (**a**) and VEGF165 (**b**) fusion proteins under different conditions. For the experiment, the fusion protein was purified by HisTrap chromatography and dialyzed against each buffer. TEV protease was treated with or without Triton X-100 and/or β-mercaptoethanol. The proteins were resolved on 4–12% Bis–Tris Plus SDS-PAGE gel. S: soluble fraction (after centrifugation); I: insoluble fraction; S + I: soluble and insoluble fraction mixture before centrifugation. The image is representative of two independent experiments

## Data Availability

All data generated or analyzed during this study are included in this published article and its supplementary information files.

## Abbreviations

aFGF: Acidic fibroblast growth factor
bFGF: Basic fibroblast growth factor
DTT: Dithiothreitol
EGF: Epidermal growth factor
FGF: Fibroblast growth factor
GF: Growth factor
HDF: Human dermal fibroblast
hGH: Human growth hormone
HPLC: High-performance liquid chromatography
HUVEC: Human umbilical vein endothelial cells
IEX: Ion exchange
IGF-1: Insulin-like growth factor 1
KGF-1: Keratinocyte growth factor 1
LB: Luria–Bertani
LC–MS/MS: Liquid chromatography-tandem mass spectrometry
MBP: Maltose-binding protein
PAGE: Polyacrylamide gel electrophoresis
PBS: Phosphate-buffered saline
PDI: Protein disulfide isomerase
PGF: Placental growth factor
RP HPLC: Reversed-phase high-performance liquid chromatography
SCF: Stem cell factor
SDS: Sodium dodecyl sulfate
TEV: Tobacco etch virus
TIMP-1: Tissue inhibitor of metalloproteinase 1
VEGF: Vascular endothelial growth factor

## References

[1] Nguyen AN, Song JA, Nguyen MT, Do BH, Kwon GG, Park SS (2017). Prokaryotic soluble expression and purification of bioactive human fibroblast growth factor 21 using maltose-binding protein. Sci Rep..

[2] Lee K, Silva EA, Mooney DJ (2010). Growth factor delivery-based tissue engineering: general approaches and a review of recent developments. J R Soc Interface.

[3] Barrientos S, Stojadinovic O, Golinko MS, Brem H, Tomic-Canic M (2008). Growth factors and cytokines in wound healing. Wound Repair Regen..

[4] Niu Y, Li Q, Ding Y, Dong L, Wang C (2018). Engineered delivery strategies for enhanced control of growth factor activities in wound healing. Adv Drug Deliv Rev.

[5] Lin WH, Xiang LJ, Shi HX, Zhang J, Jiang LP, Cai PT (2015). Fibroblast growth factors stimulate hair growth through β-catenin and Shh expression in C57BL/6 mice. Biomed Res Int.

[6] Hui Q, Jin Z, Li X, Liu C, Wang X (2018). FGF family: from drug development to clinical application. Int J Mol Sci.

[7] Li J, Yang Z, Li Z, Gu L, Wang Y, Sung C (2014). Exogenous IGF-1 promotes hair growth by stimulating cell proliferation and down regulating TGF-β1 in C57BL/6 mice *in vivo*. Growth Horm IGF Res..

[8] Walsh G (2007). Pharmaceutical biotechnology: concepts and applications.

[9] Schneider BP, Sledge GW (2007). Drug insight: VEGF as a therapeutic target for breast cancer. Nat Clin Pract Oncol.

[10] Gibbs JB (2000). Anticancer drug targets: growth factors and growth factor signaling. J Clin Invest..

[11] Ha JH, Kim HN, Moon KB, Jeon JH, Jung DH, Kim SJ (2017). Recombinant human acidic fibroblast growth factor (aFGF) expressed in *Nicotiana benthamiana* potentially inhibits skin photoaging. Planta Med.

[12] Yang J, Guan L, Guo Y, Du L, Wang F, Wang Y (2015). Expression of biologically recombinant human acidic fibroblast growth factor in *Arabidopsis thaliana* seeds via oleosin fusion technology. Gene.

[13] Żerańska J, Pasikowska M, Szczepanik B, Mlosek K, Malinowska S, Dębowska RM (2016). A study of the activity and effectiveness of recombinant fibroblast growth factor (Q40P/S47I/H93G rFGF-1) in anti-aging treatment. Postepy Dermatol Alergol..

[14] Choi SW, Pangeni R, Jung DH, Kim SJ, Park JW (2018). Construction and characterization of cell-penetrating peptide-fused fibroblast growth factor and vascular endothelial growth factor for an enhanced percutaneous delivery system. J Nanosci Nanotechnol.

[15] Kumagai Y, Kikuchi T, Nonaka A, Hiraide M, Sato S, Sakuraoka M (2019). Site-directed mutagenesis of cysteine to serine residues affects heparin binding and mitogenicity in fibroblast growth factor 4 produced in *Escherichia coli*. Biotechnol Equip..

[16] Taktak-BenAmar A, Morjen M, Mabrouk HB, Abdelmaksoud-Dammak R, Guerfali M, Fourati-Masmoudi N (2017). Expression, purification and functionality of bioactive recombinant human vascular endothelial growth factor VEGF 165 in *E. coli*. AMB Express..

[17] Nguyen MT, Krupa M, Koo B-K, Song J-A, Vu TTT, Do BH (2016). Prokaryotic soluble overexpression and purification of human VEGF165 by fusion to a maltose binding protein tag. PLoS ONE.

[18] Sun C, Li Y, Taylor SE, Mao X, Wilkinson MC, Fernig DG (2015). HaloTag is an effective expression and solubilisation fusion partner for a range of fibroblast growth factors. PeerJ..

[19] Nasiri M, Babaie J, Amiri S, Azimi E, Shamshiri S, Khalaj V (2017). SHuffle™ T7 strain is capable of producing high amount of recombinant human fibroblast growth factor-1 (rhFGF-1) with proper physicochemical and biological properties. J Biotechnol.

[20] Li D, Fu G, Tu R, Jin Z, Zhang D (2019). High-efficiency expression and secretion of human FGF21 in *Bacillus subtilis* by intercalation of a mini-cistron cassette and combinatorial optimization of cell regulatory components. Microb Cell Fact.

[21] Blaimauer K, Watzinger E, Erovic BM, Martinek H, Jagersberger T, Thurnher D (2006). Effects of epidermal growth factor and keratinocyte growth factor on the growth of oropharyngeal keratinocytes in coculture with autologous fibroblasts in a three-dimensional matrix. Cells Tissues Organs..

[22] Mohseni N, Jahanian-Najafabadi A, Kazemi-Lomedasht F, Arezomand R, Habibi-Anbouhi M, Shahbazzadeh D (2016). Recombinant expression and purification of functional vascular endothelial growth factor-121 in the baculovirus expression system. Asian Pac J Trop Med..

[23] Wu X, Yin Z, Cao C, Huang L, Lu X, Liu J (2004). Expression of human VEGF165 in silkworm (*Bombyx mori* L.) by using a recombinant baculovirus and its bioactivity assay. J Biotechnol..

[24] Wang F, Wang R, Wang Y, Zhao P, Xia Q (2015). Large-scale production of bioactive recombinant human acidic fibroblast growth factor in transgenic silkworm cocoons. Sci Rep..

[25] Rosano GL, Ceccarelli EA (2014). Recombinant protein expression in *Escherichia coli*: advances and challenges. Front Microbiol..

[26] Berkmen M (2012). Production of disulfide-bonded proteins in *Escherichia coli*. Protein Expr Purif.

[27] Ke N, Berkmen M (2014). Production of disulfide-bonded proteins in *Escherichia coli*. Curr Protoc Mol Biol..

[28] Lobstein J, Emrich CA, Jeans C, Faulkner M, Riggs P, Berkmen M (2012). SHuffle, a novel *Escherichia coli* protein expression strain capable of correctly folding disulfide bonded proteins in its cytoplasm. Microb Cell Fact.

[29] Zhao Q, Xu W, Xing L, Lin Z (2016). Recombinant production of medium-to large-sized peptides in *Escherichia coli* using a cleavable self-aggregating tag. Microb Cell Fact.

[30] Kaplan O, Zárubová J, Mikulová B, Filová E, Bártová J, Bačáková L (2016). Enhanced mitogenic activity of recombinant human vascular endothelial growth factor VEGF121 expressed in *E. coli* origami B (DE3) with molecular chaperones. PLoS ONE.

[31] Ki MR, Pack SP (2020). Fusion tags to enhance heterologous protein expression. Appl Microbiol Biotechnol.

[32] Costa SJ, Almeida A, Castro A, Domingues L, Besir H (2013). The novel Fh8 and H fusion partners for soluble protein expression in *Escherichia coli*: a comparison with the traditional gene fusion technology. Appl Microbiol Biotechnol.

[33] Silva E, Castro A, Lopes A, Rodrigues A, Dias C, Conceição A (2014). A recombinant antigen recognized by *Fasciola hepatica*-infected hosts. J Parasitol.

[34] Fraga H, Faria TQ, Pinto F, Almeida A, Brito RM, Damas AM (2010). FH8–a small EF-hand protein from Fasciola hepatica. FEBS J.

[35] Costa S, Almeida A, Castro A, Domingues L (2014). Fusion tags for protein solubility, purification and immunogenicity in *Escherichia coli*: the novel Fh8 system. Front Microbiol..

[36] Kim YS, Karisa N, Jeon WY, Lee H, Kim YC, Ahn J (2019). High-level production of N-terminal pro-brain natriuretic peptide, as a calibrant of heart failure diagnosis, in *Escherichia coli*. Appl Microbiol Biotechnol.

[37] Sun C, Liang J, Shi R, Gao X, Zhang R, Hong F (2012). Tobacco etch virus protease retains its activity in various buffers and in the presence of diverse additives. Protein Expr Purif.

[38] Holmes DI, Zachary I (2005). The vascular endothelial growth factor (VEGF) family: angiogenic factors in health and disease. Genome Biol.

[39] Jih YJ, Lien WH, Tsai WC, Yang GW, Li C, Wu LW (2001). Distinct regulation of genes by bFGF and VEGF-A in endothelial cells. Angiogenesis.

[40] Kapust RB, Tözsér J, Copeland TD, Waugh DS (2002). The P1′ specificity of tobacco etch virus protease. Biochem Biophys Res Commun.

[41] Sequeira AF, Turchetto J, Saez NJ, Peysson F, Ramond L, Duhoo Y (2017). Gene design, fusion technology and TEV cleavage conditions influence the purification of oxidized disulphide-rich venom peptides in *Escherichia coli*. Microb Cell Fact.

[42] Markert Y, Köditz J, Ulbrich-Hofmann R, Arnold U (2003). Proline versus charge concept for protein stabilization against proteolytic attack. Protein Eng.

[43] Waugh DS (2011). An overview of enzymatic reagents for the removal of affinity tags. Protein Expr Purif.

[44] Pesarrodona M, Unzueta U, Vázquez E, Clifton NJ (2015). Dialysis: a characterization method of aggregation tendency. Methods in Molecular Biology.

[45] Lebendiker M, Danieli T (2014). Production of prone-to-aggregate proteins. FEBS Lett.

[46] Kumar CS, Swamy MJ (2017). Differential modulation of the chaperone-like activity of HSP-1/2, a major protein of horse seminal plasma by anionic and cationic surfactants. Int J Biol Macromol.

[47] Banga AK (2015). Therapeutic peptides and proteins: formulation, processing, and delivery systems.

[48] Bondos SE, Bicknell A (2003). Detection and prevention of protein aggregation before, during, and after purification. Anal Biochem.

