# Supplementary material for: Effective production of human growth factors in Escherichia coli by fusing with small protein 6HFh8
Source: Microb Cell Fact. 2021 Jan 7;20:9. doi: 10.1186/s12934-020-01502-1 (PMC7791764; doi:10.1186/s12934-020-01502-1)
Supplement: Supplementary file 1 — Additional file 1: Table S1. Structural properties and molecular masses of growth factors overproduced in the current study. ‒, the range of specific region (heparin binding region in the structural properties) and protein without signal peptide and pro-region; ↔, the binding position of each residue. [file 12934_2020_1502_MOESM1_ESM.docx]

**Table S1.** Structural properties and molecular masses of growth factors overproduced in the current study. ‒, the range of specific region (heparin binding region in the structural properties) and protein without signal peptide and pro-region; ↔, the binding position of each residue.

| **Protein** | **UniProtKB** | **No. disulfide bonds (no. Cys)** | **Structural properties** | **Molecular mass (kDa), no. amino acids (protein region)** |
| --- | --- | --- | --- | --- |
| aFGF | P05230 | 0 (3) | 127‒143: heparin binding | 15.8, 140 (16‒155) |
| bFGF | P09038 | 0 (4) | 261‒277: heparin binding | 16.4, 146 (143‒288) |
| EGF | P01133 | 3 (6) | 976↔990/984↔1001/1003↔1012: Disulfide bond (intrachain) | 6.3, 53 (971‒1023) |
| hGH | P01241 | 2 (4) | 79↔191/208↔215: disulfide bond (intrachain) | 22.1, 191 (27‒217) |
| IGF-1 | P05019 | 3 (6) | 54↔96/66↔109/95↔100: disulfide bond (intrachain) | 7.7, 70 (49‒118) |
| KGF-1 | P21781 | 0 (5) | 45: *N*-linked glyosylation | 18.9, 163 (32‒194) |
| VEGF165 | P15692-4 | 5 (16) | 52↔94/83↔128/87↔130: disulfide bond (intrachain)  77↔77/86↔86: disulfide bond (interchain)  101: *N*-linked asparagine | 19.2, 165 (27–191) |
| PGF | P49763 | 5 (10) | 33/101: *N*-linked asparagine  52↔94/83↔128/87↔130: disulfide bond (intrachain)  77↔77/86↔86: disulfide bond (interchain) | 22.8, 203 (19‒221) |
| SCF | P21583 | 2 (4) | 29↔114/68↔163: disulfide bond  90/118/145: *N*-linked asparagine  167: *N*-linked serine  168/180: *N*-linked threonine | 18.5, 165 (26‒190) |
| TIMP-1 | P01033 | 6 (12) | 24↔93/26↔122/36↔147/150↔197/155↔160/168↔189: disulfide bond (intrachain)  53/101: *N*-linked asparagine | 20.7, 184 (24–207) |
