# Supplementary material for: Effective production of human growth factors in Escherichia coli by fusing with small protein 6HFh8
Source: Microb Cell Fact. 2021 Jan 7;20:9. doi: 10.1186/s12934-020-01502-1 (PMC7791764; doi:10.1186/s12934-020-01502-1)
Supplement: Supplementary file 2 — Additional file 2: Figure S1. Enhancement of TEV protease cleavage efficiency by modification of the N-terminus of the protein of interest. Glycine was inserted between the TEV protease cleavage site and N-terminus of bFGF, the protein of interest. The fusion protein was purified by HisTrap chromatography and cleaved by the TEV protease. The protein mixtures were resolved on 4–12% Bis–Tris Plus SDS-PAGE gel. The image is representative of two independent experiments [file 12934_2020_1502_MOESM2_ESM.docx]

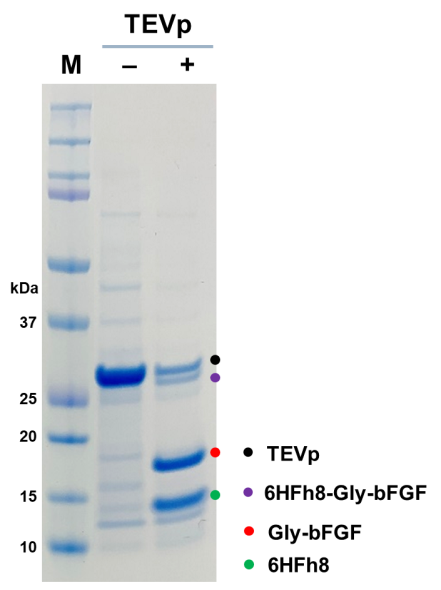


**Figure S1.** Enhancement of TEV protease cleavage efficiency by modification of the N-terminus of the protein of interest. Glycine was inserted between the TEV protease cleavage site and N-terminus of bFGF, the protein of interest. The fusion protein was purified by HisTrap chromatography and cleaved by the TEV protease. The protein mixtures were resolved on 4–12% Bis-Tris Plus SDS-PAGE gel. The image is representative of two independent experiments.
