# Supplementary material for: Effective production of human growth factors in Escherichia coli by fusing with small protein 6HFh8
Source: Microb Cell Fact. 2021 Jan 7;20:9. doi: 10.1186/s12934-020-01502-1 (PMC7791764; doi:10.1186/s12934-020-01502-1)
Supplement: Supplementary file 3 — Additional file 3: Figure S2. TEV protease treatment of aFGF (a) and VEGF165 (b) fusion proteins under different conditions. For the experiment, the fusion protein was purified by HisTrap chromatography and dialyzed against each buffer. TEV protease was treated with or without Triton X-100 and/or β-mercaptoethanol. The proteins were resolved on 4–12% Bis–Tris Plus SDS-PAGE gel. S: soluble fraction (after centrifugation); I: insoluble fraction; S + I: soluble and insoluble fraction mixture before centrifugation. The image is representative of two independent experiments [file 12934_2020_1502_MOESM3_ESM.docx]

**Figure S2.** TEV protease treatment of aFGF (**a**) and VEGF165 (**b**) fusion proteins under different conditions. For the experiment, the fusion protein was purified by HisTrap chromatography and dialyzed against each buffer. TEV protease was treated with or without Triton X-100 and/or β-mercaptoethanol. The proteins were resolved on 4–12% Bis-Tris Plus SDS-PAGE gel. S, soluble fraction (after centrifugation); I, insoluble fraction; S+I, soluble and insoluble fraction mixture before centrifugation. The image is representative of two independent experiments.
